# A hackathon as a tool to enhance research and practice on electronic health record systems’ interoperability for chronic disease management and prevention

**DOI:** 10.3389/fdgth.2023.1275711

**Published:** 2023-11-14

**Authors:** Emmanouil S. Rigas, Stavros Kostomanolakis, Nikolaos Kyriakoulakos, Dimitrios Kounalakis, Ioannis Petrakis, Alexander Berler, Asimina Boumpaki, Haralampos Karanikas, Athanasios Kelepouris, Panagiotis D. Bamidis, Dimitrios G. Katehakis

**Affiliations:** ^1^ Lab of Medical Physics and Digital Innovation, School of Medicine, Aristotle University of Thessaloniki, Thessaloniki, Greece; ^2^ Center for eHealth Applications and Services, Institute of Computer Science, FORTH, Heraklion, Greece; ^3^ Apollo SA, Athens, Greece; ^4^ MedSite, Crete, Greece; ^5^ Gnomon Informatics SA, Thessaloniki, Greece; ^6^ eHealth Services Department, Ministry of Health, Athens, Greece; ^7^ Department of Computer Science and Biomedical Informatics, University of Thessaly, Lamia, Greece; ^8^ Ministry of Health, Athens, Greece

**Keywords:** hackathon, digital health, interoperability, electronic health records (EHR), application

## Abstract

**Objectives:**

The development of a standardized technical framework for exchanging electronic health records is widely recognized as a challenging endeavor that necessitates appropriate technological, semantic, organizational, and legal interventions to support the continuity of health and care. In this context, this study delineates a pan-European hackathon aimed at evaluating the efforts undertaken by member states of the European Union to develop a European electronic health record exchange format. This format is intended to facilitate secure cross-border healthcare and optimize service delivery to citizens, paving the way toward a unified European health data space.

**Methods:**

The hackathon was conducted within the scope of the X-eHealth project. Interested parties were initially presented with a representative clinical scenario and a set of specifications pertaining to the European electronic health record exchange format, encompassing Laboratory Results Reports, Medical Imaging and Reports, and Hospital Discharge Reports. In addition, five onboarding webinars and two professional training events were organized to support the participating entities. To ensure a minimum acceptable quality threshold, a set of inclusion criteria for participants was outlined for the interested teams.

**Results:**

Eight teams participated in the hackathon, showcasing state-of-the-art applications. These teams utilized technologies such as Health Level Seven—Fast Healthcare Interoperability Resources (HL7 FHIR) and Clinical Document Architecture (CDA), alongside pertinent IHE integration profiles. They demonstrated a range of complementary uses and practices, contributing substantial inputs toward the development of future-proof electronic health record management systems.

**Conclusions:**

The execution of the hackathon demonstrated the efficacy of such approaches in uniting teams from diverse backgrounds to develop state-of-the-art applications. The outcomes produced by the event serve as proof-of-concept demonstrators for managing and preventing chronic diseases, delivering value to citizens, companies, and the research community.

## 1. Introduction

In Europe, chronic diseases are the main contributors to morbidity and mortality (1). Chronic conditions often have a sluggish, protracted course of development and are fatal. They have increased the load on health systems and caused significant human misery. Their impact is immense: in Europe, chronic diseases account for 86% of all deaths, or 4 million annually and they account for 70%–80% of total healthcare costs in the European Union (EU), or over €700 billion. In addition, many people are completely unable to work due to chronic illnesses, and nearly a quarter of those who do work (i.e., ≈23.5%) suffer from a chronic condition. Therefore, the cost of disease-related absenteeism to the EU’s gross domestic product (GDP) is projected to be 2.5% yearly.^1^ It is still difficult to determine the precise quantity, distribution, and type of the chronic disease burden in Europe. Worldwide, the frequency of chronic illnesses is increasing as populations age, and medical advancements make it possible for people with conditions that were formerly deadly to live. The effects on healthcare systems and the society at large are significant, as addressing the burden of chronic disease presents difficulties and they pose a sizeable burden for national economies, with associated costs estimated at up to 7% of a country’s gross domestic product (2).

The digital transformation of the healthcare domain (3) is among the top priorities of the EU agenda. Nevertheless, the lack of interoperability is a persistent barrier to the deployment of digital health services (4, 5). The Commission Recommendation on a European Electronic Health Record exchange format (EEHRxF)^2^ set out the framework for the development of a EEHRxF in order to achieve secure, interoperable, cross-border access to, and, exchange of, electronic health data in the EU. Furthermore, the recent proposal for a regulation for the European Health Data Space (EHDS)^3^ includes specific provisions to assist interoperability of EHR systems and of other products transmitting data to electronic health records, including medical devices, AI systems, and wellness applications. The European Health Data Space is a health-specific ecosystem comprised of rules, common standards and practices, infrastructures, and a governance framework that aims at (a) empowering individuals through increased digital access to and control of their electronic personal health data and, at (b) providing a consistent, trustworthy, and efficient set-up for the use of health data for research, innovation, policy-making, and regulatory activities.

In this context, the X-eHealth project^4^ intended to reach a common understanding in the EU on the efforts needed to adopt the commonly defined EEHRxF specifications at different levels and within national EHR solutions in member states. Focus was paid, amongst others, in the definition, specification, and demonstration of the EEHRxF use cases to support cross-border exchange of laboratory results, medical imaging and reports, hospital discharge reports, and patient summary for those suffering from rare disease and/or comorbidities to elaborate the roadmap for future uptake on the eHealth Digital Service Infrastructure (eHDSI) as well as for the additional usage within member states on the national, regional, or local level.

Recognizing the escalating need for innovative solutions and skill development in the fast-evolving tech landscape, hackathons have emerged as vital events for learning and creating. Hackathons serve as dynamic platforms where individuals, often with varied skill sets and backgrounds, collaboratively work on software or hardware projects, usually in a constrained time frame (6–8). They foster innovation, creativity, and problem-solving, allowing participants to tackle real-world issues or to explore new technologies and ideas. Hackathons are crucial in the tech ecosystem because they encourage the rapid development of prototype solutions and help in discovering and nurturing talents. They offer a space where individuals can learn, network, and collaborate, enabling the cross-pollination of ideas and the development of groundbreaking solutions, often leading to entrepreneurial initiatives and start-ups. In addition, they provide companies and organizations an opportunity to identify potential talents and innovative ideas that can drive growth and address societal needs. Overall, hackathons have shown to be efficient (9) in extracting interesting results in emerging technologies in the wider technology field. It has been adapted in taking place online (10) and has proven efficient in driving collaboration between academia and industry (11).

Within the scope of the X-eHealth project, a specialized hackathon was strategically organized, aiming to spotlight advanced applications in chronic disease management, with a special emphasis on technologies that enhance interoperability. This Hackathon intended to serve as a significant element of ongoing work, concentrating on the effective use of a variety of state-of-the-art specifications, such as HL7 FHIR, which is a standard that defines how healthcare information can be exchanged between different computer systems regardless of how it is stored in those systems, or HL7 CDA, which provides essential implementer guidance to continuously expand interoperability for clinical information shared via structured clinical notes. The event also aimed to address multilayered challenges in healthcare, such as disparities in terminologies and the intricacies of multilingualism toward promoting innovative applications of EEHRxF, elevating awareness, enriching capacity, and spreading knowledge throughout Europe. Particular emphasis was placed on the efficient exchange of electronic health records, leveraging specifications developed by the X-eHealth project, aligned with upcoming regulations for the EHDS.

Through the organization, execution, and debriefing of the hackathon that is presented in the next sections, it becomes evident that such events have the ability to help toward the specifications’ maturity. Moreover, the number and the quality of the participating teams and the solutions that were presented made it clear that the community has a high interest in this problem and that engineers and scientists with high levels of expertise exist.

## 2. Methods

The aim of the described work is to highlight, contribute, and support the European eHealth interoperability and the implementation of the EEHRxF through standardization and harmonization of health data, by raising quality and safety toward empowering citizens, healthcare professionals, and institutions. As such, following the development of the appropriate clinical scenario, upon which relevant use cases were selected for demonstrating the proof of concept, the technologies recommended for use for the purposes of the proof of concept were detailed for the use cases under validation. Assessment criteria as well as the methodological process to maximize the uptake of EEHRxF for chronic diseases management and prevention were introduced. The approach of providing detailed specifications to hackathon participants is commonplace in similar events and has proven to be beneficial to the participants (12, 13). Moreover, to optimize the output in terms of innovations and advancements from a hackathon, various best practices and recommendations have been formulated and should be prudently implemented (14, 15). Adopting such methodologies not only enhances the overall productivity and outcomes of the event but also ensures a structured and efficacious platform where technological and innovative solutions can burgeon. Ultimately, meticulous planning and a comprehensive framework for execution pave the way for maximizing gains and fostering a fertile environment for innovation and development during hackathons. The specifications and the methodology that was followed to organize the hackathon are detailed in the following sections.

### 2.1. Clinical scenario

An indicative clinical scenario was developed for the purposes of the proof of concept, including entry points for the use of EEHRxF. This scenario reveals several cases that the EEHRxF specifications can be used such as (1) exchange blood test results and imaging data between professionals in different settings and the patient, (2) transfer of hospital medical reports to family doctors and social services, (3) pathways for sickness certificates, and (4) vital signs’ measurements between patient and doctor. The full description of the indicative clinical scenario can be found in the hackathon’s webpage.^5^ In all cases, both mobile and standard computer devices should be included, and different software applications and systems are involved from different software providers. The clinical scenario described a challenging problem in life critical domains as it is the healthcare sector.

### 2.2. European electronic health record exchange format

The Commission Recommendation on a European Electronic Health Record exchange format^6^ set out the framework for the development of an EEHRxF in order to achieve secure, interoperable, cross-border access to, and exchange of, electronic health data in the EU. Priority was explicitly focused on the exchange of patient summaries, electronic prescriptions/ dispensations, medical images and image reports, laboratory results, and hospital discharge reports. For the purposes of this work, emphasis was put on the exchange of laboratory results reports, medical imaging and reports, and hospital discharge reports.

#### 2.2.1. Laboratory result reports

Clinical laboratory results play an important role in diagnosis, treatment, and follow-up of patients. Sharing of laboratory results in cross-border health information exchange facilitates chronic disease management and prevention, and is one of the priorities for the extension of eHDSI.

**Figure 1:**
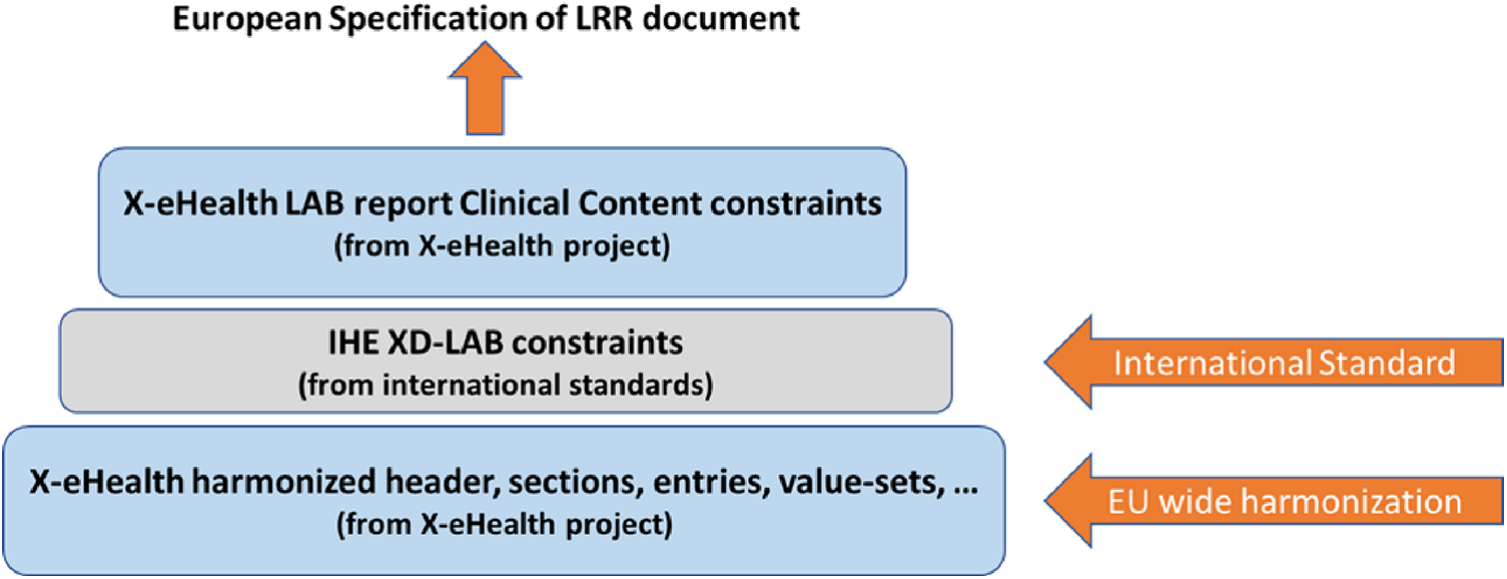
Clinical lab content standardization process for LRR document.

From the business analysis of the laboratory domain, several general use cases have been considered, each of them having a different level of priority. For the purposes of the hackathon, the querying of lab results and the laboratory results report use cases were found to be the most relevant for the provided indicative clinical scenario.^7^ Furthermore, a modeling approach that is based on the clinical information modeling standards as described by ISO/ TS 13972:2022^8^ is adopted to define the information model for the Laboratory Result Report (LRR) document (see Figure 1). Logical laboratory information model was divided into several parts (partial models) due to its complexity. Some of the partial models are specific for the laboratory domain while other models are more general (could be reused also outside laboratory domain).

The functional specifications and clinical requirements derived from the business analysis of the laboratory domain provide the input to the implementable specifications where two different formats of the LRR document are defined: HL7 CDA R2 (required) and HL7 FHIR R4 (optional). The hackathon participants were provided with all the appropriate resources to the definitions of the Laboratory Result Report document in both formats (CDA and FHIR) with the aim to experiment with the adoption and deployment of these formats to their products and finally to facilitate exchange of such LLR documents cross-border. Specifically, the tool called ART-Decor^9^ has been used for specifying, managing, and publishing the CDA R2 Templates; and the combination of GitHub,^10^ FHIR Shorthand (FSH),^11^ FHIR Implementation Guide (IG) Publisher,^12^ and the FHIR Continuous Integration (CI)-Build site^13^ for specifying, managing, and publishing FHIR-based specifications. ART-DECOR is an open-source tool suite that supports the creation and maintenance of HL7 templates, value sets, scenarios, and data sets. It is an established tool to manage the specifications and it was also used by the X-eHealth project.

#### 2.2.2. Medical imaging and reports

The main objective for sharing images and diagnostic reports is to allow authorized healthcare providers and patients to access the patient’s imaging records from any types of systems (EMR, EHR, PACS, mobile applications, etc.) stored in several repositories belonging to one or more affinity domains.

To describe the different common elements that are part of the Diagnostic Imaging Report, certain information models have been developed to reduce the complexity of the business process architecture and provide support for informed decision making. These information models allow mapping functional specifications to HL7 CDA^14^ and contain the elements grouped in different information packages (known as modules), which can be optional or mandatory depending on the medical needs of the healthcare institutions as well as the data available to prepare the hospital discharge report.

**Figure 2:**
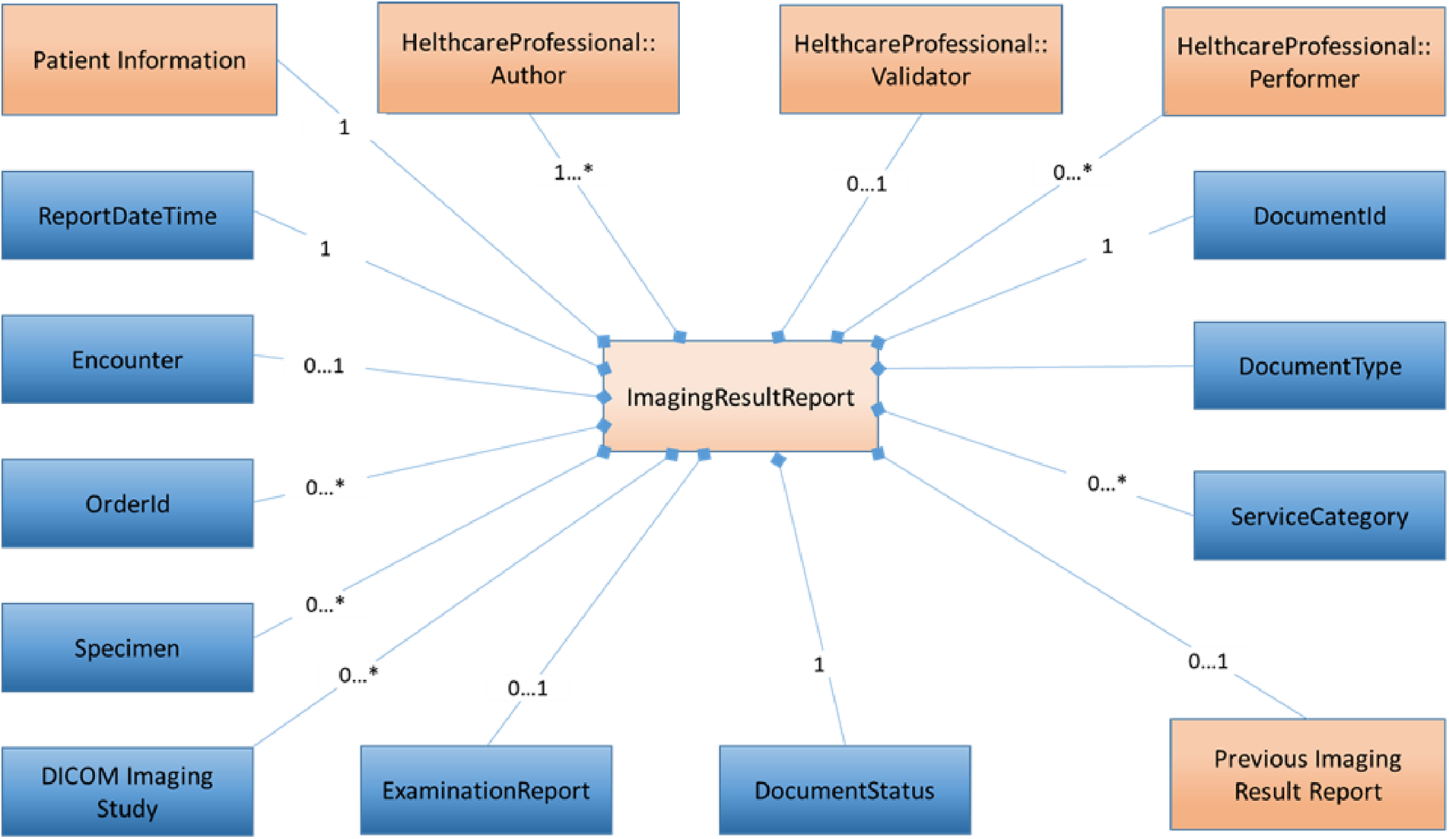
X-eHealth information model.

The information model^15^ for Medical Imaging and Imaging Reports is presented in Figure 2. This information model is compatible with the Diagnostic Imaging report using DICOM part 20^16^ as the basis of the specification for the X-eHealth IG Diagnostic Imaging Report developed in Art Décor.^17^ DICOM part 20 (Imaging reports using HL7 CDA) considered multiple layers of constraints from HL7v3 RIM, CDA r2, DICOM, and professional contents for specialized procedures. The relationships between X-eHealth information model, HL7 CDA, and DICOM part 20 are presented in the X-eHealth implementation guide for diagnostic imaging report.

As far as mapping functional specifications to HL7 FHIR is concerned, FHIR diagnostic report^18^ regroups the resources about the diagnostic report itself and about the subject. It provides a combination of request information, atomic results, images, interpretation, and formatted reports. As described above for DICOM part 20, the FHIR Diagnostic report is constructed in the same way: from imaging studies on the top of the hierarchy, to series of images in the middle, and to instances at the lower level.

FHIR resources are specified in the FHIR IG.^19^ The IG helps solve problems and clarify the use of the resources providing a human-readable part as well as a set of computable conformance resources. FHIR defines the resource ImagingStudy,^20^ which provides information on a DICOM imaging study, and the series and imaging objects in that study. It also provides information on how to retrieve that information in a native DICOM format, or in a rendered format, such as JPEG. FHIR also defines the resource DiagnosticReport,^21^ which includes clinical context such as requesting and provider information, and some mix of atomic results, images, textual and coded interpretations, and formatted representation of diagnostic reports.

#### 2.2.3. Hospital discharge reports

The main information model for Hospital Discharge Report (HDR) consists of several concepts that cover different types of information, some of them being containers (modules) of other concepts. The model is composed of “containers,” which are the main structural units produced to represent the logical hierarchy of an information system, from which all other components are derived. Relationships may exist between two or more containers indicating that at least one is a sub-component of the other (e.g., the “Address” container may be associated with the “Patient” container, as the patient is always associated with some address). To implement the designed HDR information model, a set of candidate HL7 CDA templates and FHIR profiles have been selected among those defined and used in existing standardized Hospital Discharge Result Report or related documents.

The aim of the information models is to help define the detailed structure. It is essentially a non-exhaustive “list” of key components organized into a hierarchical structure that reflects the varying relationships among them. Individual models reuse other modules (formalized as Information Models); each module describes different aspects of common parts of the X-eHealth documents, e.g., the detail of the hospital encounter documented by the HDR. The HDR information model (see Figure 2) is the entry point for the HDR document.^22^

**Figure 3:**
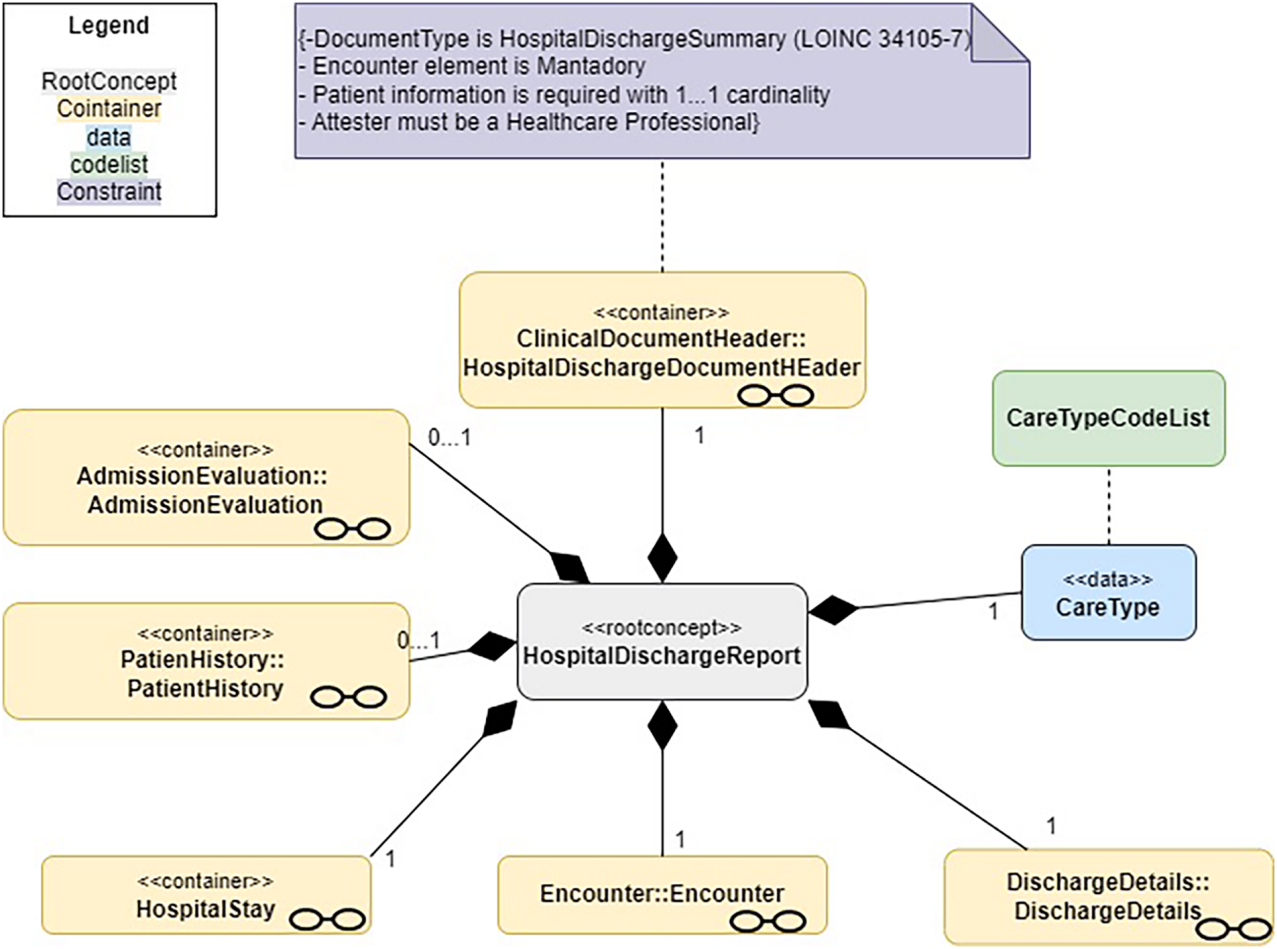
A logical model of the common EU hospital discharge report.

To implement the designed HDR information model (see Figure 3), a set of candidate section templates have been selected among those defined and used in existing standardized Discharge Summaries or related documents. For the HL7 FHIR implementation, general considerations similar to those done for the HL7 CDA implementation have been done. A set of tools used in order to generate FHIR human-readable IG and the computable FHIR package can be published in a public site.

### 2.3. Recommended technologies

Initial foreseen technologies have been selected and introduced to the hackathon participants, in order to support standardized and secure exchange of EHR information in their introduced proof-of-concept. These technologies are briefly presented below:

• HL7 FHIR: Healthcare data can be shared across various computer systems independent of how they are kept in those systems according to the HL7 FHIR standard. This makes it possible for clinical and administrative data to be securely accessible to those who require it and to those who have the right to do so on behalf of a patient receiving care. A collection of modular parts known as “Resources” is used to construct FHIR solutions. These materials can be quickly combined into functional systems that, for a small fraction of the cost of current options, address actual clinical and administrative issues. FHIR is suitable to be used in a wide variety of contexts, such as mobile apps, cloud communications, EHR-based data sharing, and server communication in large institutional healthcare providers. The X-eHealth FHIR IG provides a human-readable representation as a browsable website and a list of artifacts including examples of Logical Models, Resource Profiles, Extension Definitions, and Terminologies. The on-development X-eHealth FHIR IG will be published in the build.fhir.org site.^23^
• HL7 CDA: In order to facilitate the transmission of “clinical papers” between healthcare professionals and patients, HL7 developed the Clinical Document Architecture, which is a document markup standard. According to its definition, a clinical document must possess the following six characteristics: (i) persistence, (ii) stewardship, (iii) potential for authentication, (iv) context, (v) wholeness, and (vi) human readability. X-eHealth CDA specifications are published in the Art Decor X-eHealth project environment.^24^ As mentioned before, Art Decor is an open-source tool suite that supports the creation and maintenance of HL7 templates, value sets, scenarios, and data sets. The tool features cloud-based federated Building Block Repositories (BBR) for templates and value sets.
• IHE XDS: This Cross-Enterprise Document Sharing profile explains how to go about sharing medical data electronically with peers.
• IHE MHD: In order to make the deployment of mobile apps more consistent and reusable, the Mobile access to Health Documents (MHD) Profile defines one standardized interface to health document sharing [i.e., an Application Programming Interface (API)] for usage by mobile devices.
• IHE XDR-I: Using a dependable messaging system, Cross-Enterprise Document Reliable Interchange of Images (XDR-I) offers DICOM service-object pair (SOP) instances and image reports. This enables direct imaging document exchange between a source of imaging documents and other healthcare IT systems that support imaging documents.
• IHE XDR: The purpose of Cross-Enterprise Document Reliable Interchange (XDR) is to serve as a quick, point-to-point introduction to XDS. To transfer documents and related metadata between two systems, it uses the Provide and Register Document Set transaction, which was first specified in XDS.
• IHE XDM: Cross-Enterprise Document Media Interchange (XDM) enables document exchange over a variety of common media types using a common file and directory structure. This enables the patient to carry medical records on tangible media. This also makes it possible to send medical documents via personal email.

### 2.4. Capacity building

Once the scenario focused on chronic disease management was meticulously finalized and the associated specifications were carefully identified and detailed, the intricate process of preparing for the hackathon was set into motion. In this highly collaborative and informative context, several on-boarding webinars (summarized in Table 1) were orchestrated to pave the way for a successful event.

**Table 1:** Summary of webinars.

No. of webinar	Description
Webinar 1	Introduction to the hackathon and the specifications
Webinar 2	Introduction to the hackathon and the X-eHealth project. Details about specifications on Art Decor and hospital discharge reports
Webinar 3	Introduction to the hackathon and the X-eHealth project. Details about a use case related to rare diseases and specifications on lab reports
Webinar 4	Introduction to the hackathon and details about medical imaging and hospital discharge reports
Webinar 5	Preparation of the participating teams for the upcoming hackathon

The aim of hosting these webinars was multifaceted. First, they served as a communicative platform to introduce the hackathon to potential participants, elucidating the logistics and technical specifications that would guide the creations during the event. Second, they played a crucial role in drawing attention from the community by effectively communicating the event’s significance and details, thereby ensuring that participants were well-prepared to craft high-quality, innovative applications during the hackathon.

To elaborate, a series of five meticulously planned webinars were conducted. The inaugural webinar offered a comprehensive introduction to the hackathon and delineated the relevant specifications. The second one reached out to all interested stakeholders, unraveling the concept and the pressing need for the hackathon, and detailed the objectives of the X-eHealth project along with nuances of Art Decor and hospital discharge reports.

The third webinar enlightened stakeholders about a unique use case centered around rare diseases and specifications pertinent to lab reports, reinforcing the concept and aims of the hackathon and the X-eHealth project. The fourth webinar delved deeper, exploring aspects of medical imaging and hospital discharge reports.

The fifth and concluding webinar was instrumental in gearing up the participating teams for the forthcoming hackathon, with an initial focus on specifications related to HL7 FHIR, followed by illustrative examples centered around medical imaging. Subsequently, the teams enrolled for the hackathon were introduced, and the event’s agenda, the meticulously planned program spanning the three days of the hackathon, and the evaluation criteria to be employed were shared with the participants.

Concurrently, two professional training events were also conducted, titled “Sharing Laboratory Data Cross-Borders: Understanding Future Direction and Technical Solutions for Laboratory Data Exchange” and “Data Exchange for Better Health—Accessing and Sharing Medical Images and Discharge Letters Across Europe,” offering deeper insights and perspectives on the themes of the hackathon.

These webinars and training events were not merely informative sessions but were pivotal in setting the tone for the hackathon, ensuring participants were well-informed, and fostering an environment conducive to innovation and learning, ultimately contributing to the realization of the overarching goals of the X-eHealth project.

### 2.5. Assessment criteria

The assessment criteria selected for the evaluation of each of the propositions for proof-of-concept to be presented by the participants were decided to be (i) innovation and originality, (ii) impact and benefit for the patient and other stakeholders of the healthcare system, (iii) applicability and degree of implementation of functional features that have business value, (iv) feasibility and technical soundness, (v) technical background and whether a live demonstration of a running application is presented, and (vi) contribution to the EEHRxF development and feedback provided to the EEHRxF specifications. These criteria were selected based on the experience of the organizing team and the needs of the X-eHealth project. The teams that took part in the event’s final day and presented their work met the aforementioned requirements, following an initial screening process.

## 3. Results

Once all the application forms submitted from interested teams were collected, these were evaluated based on the relation of the proposed solution with the scope of the hackathon and on the technologies that were proposed to be used and whether these were to some extent matching the specifications that were provided. In total, 12 teams declared their intention to participate in the event, of which eight were selected to participate in the final event.

**Figure 4:**
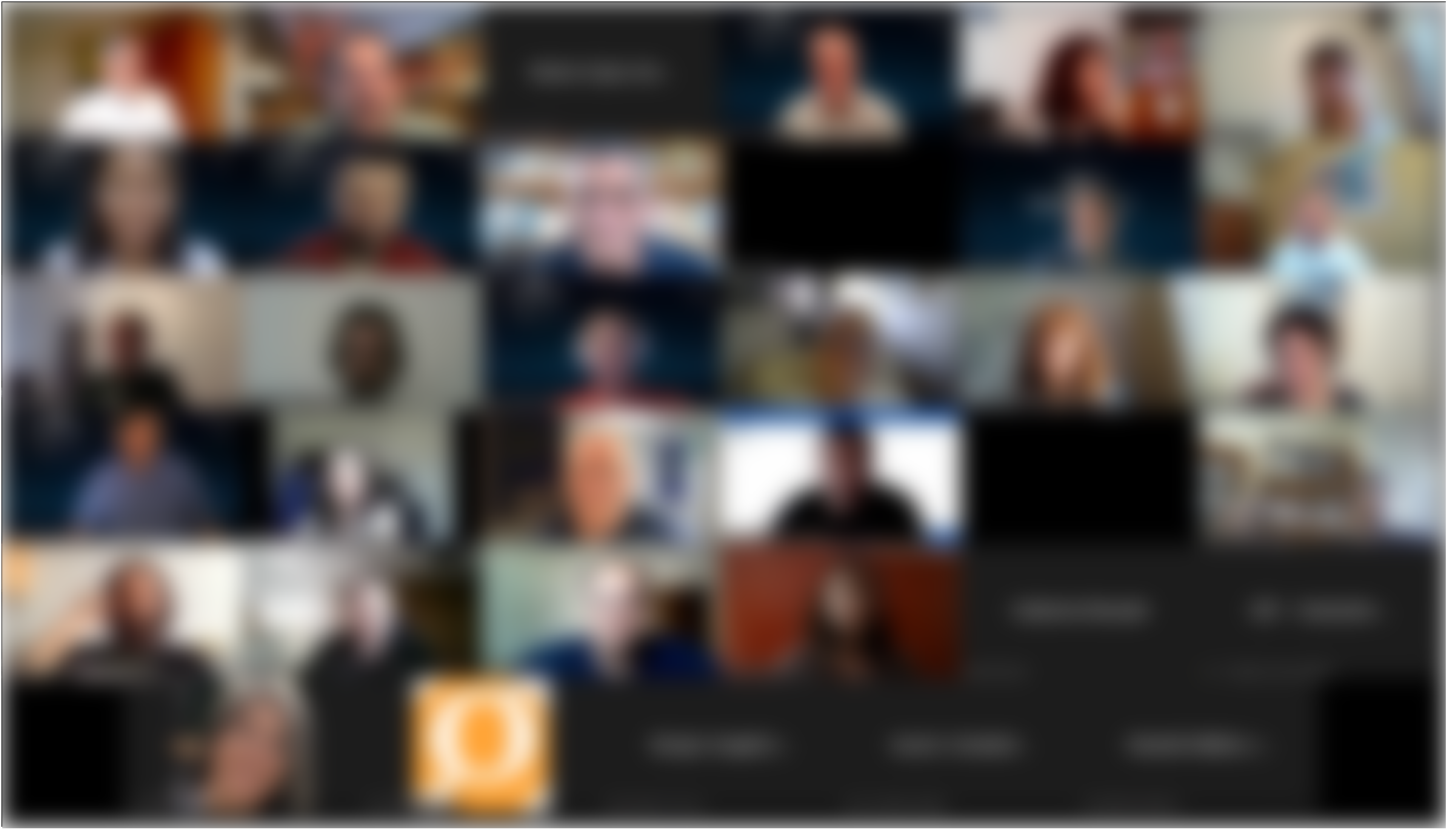
Snapshot of the hackathon event - day 3.

The proof-of-concept event took place in the form of an online hackathon between 7 and 9 June 2022 (see detailed program in Table 2 and an indicative screenshot of the event in Figure 4). The first day of the event involved a welcome message and keynote speeches by the project manager and the relevant task leader, a short introduction of each participating team, a short presentation of the available material and tools to the teams, and the presentation of the hackathon’s rules and logistics. In total, 44 participants attended this first part of the hackathon. In addition, the teams had the chance to discuss with the mentors that were assigned to them in one-to-one meetings. During the second day, informing sessions regarding the four specifications were organized and they were open to be attended by any interested participant. Finally, during the third and final day of the event, the presentation of the proof-of-concept applications from the participating teams took place. The evaluation of the proof of concepts and their demonstrators were used to capture and share best practices and guidelines for EEHRxF implementation.

**Table 2:** X-eHealth hackathon program and contents.

Day of hackathon	Description
Day 1: 7 June 2022	Keynote speeches by project manager and task leader. Short introduction of each team. Short presentation of available material and tools to the team. Hackathon rules and logistics.
Day 2: 8 June 2022	Ask anything special themes: Laboratory requests and reports, medical imaging and reports, refined PS for read diseases. Hospital discharge reports.
Day 3: 9 June 2022	Welcome and introduction. Recap of event and hackathon logistics. Participants’ presentations. Hackathon results announcement. MyHealth@EU current and future services. Keynote speech—The future of the European Electronic Health Record Exchange Format. Future events and closing notes from the X-eHealth Project.

### 3.1. Proofs of concept

Out of the eight teams, seven participated in the final day of the hackathon and showcased their works. Each presented work, to some extent, related to the topics provided by the X-eHealth project. The contribution of each team is described in the following paragraphs and is summarized in Table 3. Note that all the material produced during the event is available online.^25^

**Table 3:** Summary of teams that participated in the final hackathon event.

Team name	Area of application	Characteristics	Technology	Demonstration scale
DoctorNearYou	eHealth Services	Product; other eHealth Services	HL7 FHIR, HL7 CDA	Local
GNOMON-TEAM	ePrescription for medication	Prototype; product	HL7.FHIR, IHE.XDS, HL7.CDA	Regional; cross-border
HiSpin	Laboratory	Prototype	HL7-FHIR	Local
iMedPhys-AUTH	Laboratory	Prototype; deliverable in research project	HL7 FHIR	Regional
dm-RET	Telemonitoring—teleconsultation	Deliverable in research project	HL7 FHIR	Local
Integrated Care Solutions	Laboratory, discharge letters, radiology, imaging, patient summary	Product	HL7 FHIR, HL7 CDA, IHE XDS and other	Local; regional; national; cross-border
eHealth4U	Discharge letters and laboratory	Prototype; Deliverable in research project	HL7 FHIR	National

• DoctorNearYou: Proposed a platform that provides eHealth services, which are user-friendly and convenient connecting patients with Healthcare Professionals. The aim of this platform was to increase sales by driving adoption of telemedicine services in Greece among locals and tourists alike drive increase of eRx and e-appointment services with healthcare professionals. It leverages the HL7 FHIR standard in order to retrieve and store synchronized healthcare data in an interoperable, efficient, and secure way.
• GNOMON-TEAM: Proposed a solution to collect important information from the continuity of care record of a patient, considering displaying patient summaries (EU and IPS format), displaying lab reports and medical imaging reports, displaying hospital discharge letter information, and visualizing current and past medication lists. The area of application is ePrescription for medication and HL7.FHIR, IHE.XDS, HL7.CDA were used.
• HiSpin: Participated with the adoption of the X-eHEALTH format for health data exchange of clinical lab results between HiSpin’s CiviCARE Personal Health Record (PHR) and health information systems. CiviCARE PHR helps citizens and patients to store all their health information in one place and share with healthcare professionals of their choice. A key characteristic of CiviCARE is that it is by design interoperable, using a data model based on HL7-FHIR. CiviCARE intends to use additional acknowledged standards for data export and transmission so that it can communicate with health information systems and clinical laboratories provided they also adopt established standards. In the content of this hackathon, the export functionality of lab results in the X-eHEALTH format was implemented as an early prototype.
• iMedPhys-AUTH: Proposed an FHIR-based application that takes as input a laboratory report and uses an ML algorithm to predict if a patient has over 50% stenosis of the coronary artery, which leads to the need to undergo coronary computed tomography angiography (CCTA). Variables from lab results were mapped to FHIR resources before they were fed into the ML algorithms for further analysis.
• dm-RET: The aim of the proposed solution is to improve the quality of life via tele-monitoring and tele-consulting of the heart failure patient using the HL7 FHIR standard. Specifically, it aims to develop and deliver an innovative platform supporting enhanced clinical monitoring and interventions aimed at improving the clinical management of patients with chronic heart failure, reducing their mortality and hospitalization rates, and improving their quality of life, safety, and wellbeing.
• Integrated Care Solutions: Presented a suite of products that addresses the needs of patient-centric healthcare systems, in order to proactively manage clinical and administrative processes and activities. Each software module can be installed at one or more departments of a health organization. Modules are interoperable with each other and modules of third-party systems. All applications can exchange information, so that all records are stored uniformly and reviewed by all users with permission rights. At the same time, these applications can exchange information with disparate systems that support international standards facilitating national and cross-border exchange of EHR data.
• eHealth4U—Integrated National EHR System in Cyprus: The proposed application was part of the eHealth4U project that undertakes the challenge of defining the structure and the content of the national integrated EHR system in Cyprus and developing a prototype of it. Their vision is that the outcome of this project will establish the foundations of the country’s broader eHealth ecosystem adhering to the Electronic Health Law of Cyprus (No.59(I)/2019). It involves discharge letters and laboratory results.

### 3.2. Hackathon’s evaluation from the teams

Once the hackathon ended, the participants were provided with a detailed questionnaire to evaluate the event both in terms of its organization and in terms of its ability to produce insightful results. The main questions are summarized as follows and summarized in Table 4:

**Table 4:** Summary of the evaluation from the participants.

Question	Summary of replies
Did you participate in any team?	Yes: 81%, No: 19%
How old are you?	18–29: 7%, 30–39: 40%, 40–49: 46%, older than 50: 7%
How many years of working experience do you have?	<10 years: 31%, >=10 years: 69%
What is the type of your organization?	Public sector: 50%, private sector: 50%
Please select your profession	Engineers: 50%, medical informatics researchers: 19%, software developers: 13%, other: 18%
How do you rate the webinars that took place prior to the event?	Score 5: 50%, score 4: 37.5%, score 3: 12.5%
How do you rate the technical material that was provided to you by the organizers?	Score 5: 43.75%, score 4: 37.5%, score 3: 18.75%
How do you rate the support you received during the event?	Score 5: 62.5%, score 4: 25%, score 3: 12.5%
How do you rate the topic of the hackathon?	Score 5: 87.5%, score 4: 12.5%
How do you rate the feedback that you received from the evaluation committee?	Score 5: 50%, score 4: 31.25%, score 3:12.5%, score 2: 6.25%
To what extent has this hackathon helped you in developing innovative solutions?	Score 5: 62.5%, score 4: 12.5%, score 3: 12.5%, score 2:6.25%
To what extent has this hackathon helped you to expand your technical abilities and knowledge?	Score 5: 43.75%, score 4: 31.25%, score 3: 12.5%, score 2: 6.25%
How do you rate the hackathon overall?	Score 5: 43.75%, score 4: 53.25%

The following questions are related to the profile of the participants. In the question *Did you participate in any team?*, 81% of the replies were positive and 19% were negative. This means that the majority of the people that participated in the hackathon were involved in one of the teams, but participants that were just interested in the topic also existed. In the question *How old are you?*, 7% were between 18 and 29 years, 40% were between 30 and 39 years, 46% were between 40 and 49 years, and 7% were older than 50 years. From these results, we can conclude that most of the participants can be considered experienced researchers and/or practitioners. This can be supported by the fact that in the question *How many years of working experience do you have?*, 69% stated that they have more than 10 years of experience. In the question *What is the type of your organization?*, 50% of the participants stated that they come from public institutions and 50% from the private sector. This shows that there was a balance between people from academia and people from the industry. Finally, in the question *Please select your profession*, the majority were engineers (25%), medical informatics researchers (19%) and software developers (13%), while healthcare providers, laboratory technicians, data scientists, technology service providers in healthcare, researchers, and policymakers also existed.

The following questions are related to the participants’ opinion on the hackathon and are in Likert scale (1: lower score, 5: higher score). In the question *How do you rate the webinars that took place prior to the event?*, 50% scored them with 5, 37.5% scored them with 4, and 12.5% scored them with 3. In the question *How do you rate the technical material that was provided to you by the organizers?*, 43.75% scored it with 5, 37.5% scored it with 4, and 18.75% scored it with 3. In the question *How do you rate the support you received during the event?*, 62.5% scored it with 5, 25% scored it with 4, and 12.5% scored it with 3. From these three questions, we can conclude that the participants were overall satisfied from the support that was provided to them both prior and during the event.

In the question *How do you rate the topic of the hackathon?*, 87.5% scored it with 5 and 12.5% scored it with 4. In the question *How do you rate the feedback that you received from the evaluation committee?*, 50% scored it with 5, 31.25% scored it with 4, 12.5% scored it with 3, and 6.25% scored it with 2. In the question *To what extent has this hackathon helped you in developing innovative solutions?*, 62.5% scored it with 5, 12.5% scored it with 4, 12.5% scored it with 3, and 6.25% with 2. In the question *To what extent has this hackathon helped you to expand your technical abilities and knowledge?*, 43.75% scored it with 5, 31.25% scored it with 4, 12.5% scored it with 3, and 6.25% scored it with 2. Finally, in the question *How do you rate the hackathon overall?*, 43.75% scored it with 5 and 53.25% scored it with 4. From this set of questions, we can conclude that overall the participants were satisfied by the topic of the hackathon, the feedback they received, and its usefulness in expanding their skills. However, in the last two areas, some space for improvements exists.

## 4. Discussion

Medical tourism, support for continuity of care, personal health records, secondary use of data, telemonitoring and teleconsultation, hospital care, and establishment of a national EHR system were some of the areas and application types included in the suggested proof of concept applications. Target audiences for the hackathon included researchers, medical experts, the general public (patients), and national authorities. Several technologies were employed, including HL7 FHIR, IHE XDS, and HL7 CDA. The standard framework created by the X-eHealth project for medical imaging, discharge letters, lab findings, and patient summaries was implemented into the proof of concepts. We contend that the X-eHealth standards that were evaluated during the hackathon considerably aided the participating teams in developing the presented apps, even though they could not be regarded as final at the time of the event. These applications addressed the usage of EEHRxF at all levels and were based on both commercial goods and research efforts (i.e., cross-border, national, regional, and local level). Although some of the applications were not directly related to chronic diseases, they have the potential to be adapted to be used in such diseases as well.

This hackathon created a collaborative space for diverse talents, fostering the creation of impactful solutions and attracting interest for further refinement and research from various stakeholders and experts. The innovations conceived in this vibrant environment, with proper support and direction, are poised to be influential in advancing science, technology, and economic growth. The outcomes of this hackathon confirm the usefulness of such events as has been described in the literature. For example, Yarmohammadian et al. (16) describe and support the use of hackathons to educate students in health-related technological domains. Poncette et al. (14) depict hackathons as drivers for healthcare innovations and outline the cost and time effectiveness of such approaches. Finally, Wang et al. (17) praise the usefulness of hackathons to promote collaborations between researchers and practitioners from different institutions and possibly countries. In this context, we argue that this hackathon has been a catalyst for developing applications with significant scientific and practical merit, holding the potential to transform into innovative start-ups and revolutionary products (13).

On the third day of the event, a highly insightful conversation about the EEHRxF’s future was also started. It focused on the function of the X-eHealth project and how it fits into the overall context of European digital health. In order to support interoperability and data portability, which would improve individual ownership over their electronic health data, the envisioned shared EHDS establishes important specifications expressly for EHR systems. The new data domains that the X-eHealth project is concentrating on are included in the priority categories of electronic health data for primary use that are outlined in the proposed regulation.

Consequently, the consensus among the involved parties underscores the crucial necessity to integrate into both existing and emerging systems the capability to generate and interpret medical documents in a standardized format. It is also vital to utilize standardized technologies for exchanging these documents in their domestic and international interactions. The information derived is anticipated to enlighten governments, trade associations, patients, health professionals, and informal caregiver associations and will also lay the groundwork for discussions introducing new use cases of the eHN multiannual work program. The hackathon proved exceptionally useful in this context, serving as a creative and collaborative environment where diverse teams could troubleshoot, innovate, and rapidly prototype solutions. It highlighted the value of such events in accelerating the development of practical solutions, fostering cross-disciplinary collaboration, and enabling the rapid exchange of ideas and expertise.

This undertaking provided invaluable feedback essential for refining the EEHRxF’s functional and technical specifications to improve patient summaries, discharge summaries, lab results, imaging studies, and reports, especially for patients dealing with chronic diseases or comorbidities. Finally, the positive feedback received from the participating teams concerning the organization and the applicability of the hackathon has sown seeds of motivation for the conduct of similar events in the future, underscoring the invaluable role of hackathons in driving forward innovations in healthcare.

## 5. Conclusion

The absence of a finalized standard on exchanging electronic health records across the EU puts barriers in the design, development, take-up, sustainability, and exploitation of such applications that depend on availability of EHR data. Through the organization, execution, and debriefing of the hackathon that was presented in this paper, it became evident that such events have the ability to help toward the specifications’ maturity. Moreover, the number and the quality of the teams that participated in the event and the solutions that were presented made it clear that the community has high interest in this problem and that engineers and scientists with high levels of expertise exist. In addition, it proved that hackathons have the ability to produce valuable applications and insights into emerging technological areas. Thus, the successful execution of this hackathon led to the organization from the X-eHealth project of a second hackathon on Rare Diseases and Cancers.^26^ Overall, the participants that have experience in Healthcare Standards technologies that are used in the X-eHealth project (e.g., CDA, FHIR, Document Sharing infrastructure) confirmed that the emerging standards are very close to the already existing exchange formats (e.g., IPS, ePrescription, Diagnostic Report, etc.), and they felt confident that they could easily incorporate them into their systems.

While hackathons are lauded for fostering innovation and collaboration, they face several limitations including time constraints, which often result in underdeveloped prototypes. The lack of post-event support impedes the sustainability and further development of promising projects. The rapid, intense nature of these events sometimes compromises the depth and quality of solutions and can lead to participant burnout. Furthermore, diversity and inclusion challenges, intellectual property disputes, limited scope, and accessibility issues can narrow the range of solutions and hinder broader participation. Monetary constraints and lack of adequate rewards can also impact motivation, and many solutions face significant hurdles in real-world implementation due to feasibility and adaptability concerns. Balancing these limitations with effective organization, clear objectives, and participant support can elevate the overall impact and productivity of hackathons. Apart from these, the described study has the following limitations: (1) The study used a limited number of specifications that focused on specific use cases. (2) Given the provided specifications, the developed solutions were related to the interests of the project’s community of practice. (3) The emphasis of the study was on the practical aspects of developing usable solutions related to the EU’s policy on interoperability healthcare systems. The specifications were designed based on this need. (4) The methodological approach was based on the approved methodology of implementing a project^27^ for future expansion of the EEHRxF standard. (5) The aim of this study centered on the practical aspects of developing solutions to support interoperability, while scientific novelty was not the primary objective.

In terms of future work, it would be very interesting and useful to repeat such a hackathon event once the specifications have been finalized and ask from the participants to present updated solutions and to motivate them to continue the work toward a sustainable set of interoperability tools to effectively support the vast ecosystem of digital health solutions aiming toward chronic disease management and prevention (18). Moreover, the standardization of at least some of these applications, would seal the success of this hackathon. Finally, the cooperation and possible integration with the EHDS in future projects is expected to enhance the applicability of demonstrated proof of concepts developed across the EU at a global scale.

## Data Availability

The original contributions presented in the study are included in the article, further inquiries can be directed to the corresponding author.

## Footnotes

^1^https://ec.europa.eu/health/newsletter/169/focus_newsletter_en.htm.

^2^https://eur-lex.europa.eu/legal-content/EN/TXT/PDF/?uri=CELEX:32019H0243.

^3^https://health.ec.europa.eu/publications/proposal-regulation-european-health-data-space_en.

^4^https://www.x-ehealth.eu/.

^5^https://www.x-ehealth.eu/hackathon-for-chronic-disease-management/.

^6^https://eur-lex.europa.eu/legal-content/EN/TXT/PDF/?uri=CELEX:32019H0243.

^7^https://www.x-ehealth.eu/wp-content/uploads/2022/09/D5.3-Laboratory-Requests-and-Reports-guideline-and-functional-specifications.pdf.

^8^https://www.iso.org/standard/79498.html.

^9^https://wordpress.art-decor.org/.

^10^http://github.com/.

^11^https://build.fhir.org/ig/HL7/fhir-shorthand/.

^12^https://github.com/HL7/fhir-ig-publisher.

^13^https://build.fhir.org/.

^14^http://www.hl7.org/implement/standards/product_brief.cfm?product_id=7.

^15^https://www.x-ehealth.eu/wp-content/uploads/2022/09/D5.4-Medical-Imaging-and-Imaging-Reports-guideline-and-functional-specifications.pdf.

^16^https://dicom.nema.org/medical/dicom/2022e/output/pdf/part20_changes.pdf.

^17^https://art-decor.ihe-europe.net/art-decor/decor-templates–DICOM-?section=templates.

^18^https://build.fhir.org/diagnosticreport.html.

^19^https://www.hl7.org/fhir/implementationguide.html.

^20^ https://hl7.org/fhir/imagingstudy.html

^21^ https://www.hl7.org/fhir/diagnosticreport.html

^22^https://www.x-ehealth.eu/wp-content/uploads/2022/09/D5.5-%E2%80%93-Hospital-Discharge-Reports-guideline-and-functional-specifications.pdf.

^23^https://build.fhir.org/ig/hl7-eu/x-ehealth/artifacts.html.

^24^https://art-decor.org/art-decor/decor-project–eehrxf-.

^25^https://www.x-ehealth.eu/hackathon-for-chronic-disease-management/.

^26^https://www.x-ehealth.eu/hackathon-for-rare-diseases-and-cancers/.

^27^https://cordis.europa.eu/project/id/101095594.
